# Honesty and Push

**Published:** 1876-03

**Authors:** 


### *HONESTY AND PUSH.*

In an article published in our January number we alluded to the genuine enterprise manifested by Dr. Dundas Dick, the gentleman who coats fluid medicines with a film of gelatine so that they shall not offend the palate. Our remarks were merely by way of comment upon an article in the *Reporter*, a paper published by the great advertising men, G. P. Rowell & Co. A good, Christian friend of ours, a druggist by profession, calls attention to our article and reminds us that, in our well-intentioned comments on Dr. Dick's business habits, we omitted one very essential point—his sterling honesty. Our friend thinks that, ordinarily, too much credit is given to energy and business activity, and too little to those weightier qualities which go to make up the substantial character—integrity and scrupulous fair-dealing. He says that the example to be placed before men is not that of success achieved by cunning, or by push, or by notoriety; but rather that of wealth accumulated or influence gained by righteousness—that is, by right-doing.

In this our friend is correct, and we have never held to any other view. When we said that Dr. Dick's success, like the success of Mr. A. T. Stewart, resulted largely from his ability to do, better than any of his many employés can do, all the multifarious duties of his factory, we did not mean by this to indicate that his intimate practical acquaintance with the details of his vast business could be considered as taking the place of integrity. No knowledge can compensate for lack of honesty. The two must go hand in hand, or failure will come sooner or latter. A good lady, who knew Mr. Stewart when he had only one clerk, told us a few days ago, that he never allowed that clerk or any of the many thousands since and now in his employ, to recommend goods offered for sale. His plan has always been to exhibit the articles and let them do their own talking. So with Dr. Dick. Physicians know that they can prescribe his pure, protected remedies with the certainty that the result anticipated will follow their use. They are just what they purport to be, and nothing more nor less. It is as if he were to seal up pure oils and balsams and terabinthenates in transparent glass, so far as purity is concerned. The only difference is that the glass is soluble and nutritious, being made of transparent gelatine.

Let us remark that Dr. Dick is not a manufacturer of "patent medicines." His tasteless remedies are not secret or proprietary nostrums. They are strictly "officinal;" that is, they are the authorized medicines of the United States Dispensatory. It is only in selecting pure, fresh drugs, and protecting them with the pure gelatinous film, rapidly and elegantly, that Dr. Dick's great skill is shown. The drugs thus inclosed are potent, active, powerful, and are usually ordered by physicians. Druggists of our acquaintance testify that more than three-fourths of all of Dr. Dick's goods sold are called for by written prescriptions. When thus ordered the druggist removes the outer wrapper and only the magic letters "D. D. & Co." denote the maker's name.

Dr. Dick has earned his popularity by fair-dealing. The products of no chemist's labratory stand higher than his. He has, by the integrity of his methods and the purity of his medicines, placed himself on the plane occupied by Dr. Squibb of Brooklyn, whose manufactured drugs stand at the head. If knowledge and energy and good taste have done much of this, honesty and integrity have done even more. The druggists and the doctors all know that Dundas Dick's tasteless medicines are to be relied upon. In the one word—faithfulness—we have the causes of his vast success clearly summed up.

